# Depression and substance abuse among university students

**DOI:** 10.1097/MD.0000000000041671

**Published:** 2025-02-28

**Authors:** Kefie Manaye Mengistie, Kelemu Zelalem Berhanu

**Affiliations:** a Department of Psychology, Debre Markos University, Debre Markos, Ethiopia; b University of Johannesburg, Johannesburg, South Africa.

**Keywords:** alcohol, depression, khat, substance abuse, university students

## Abstract

The purpose of the study was to examine the association between depression and substance abuse and to assess their prevalence and associated factors. A cross-sectional design was employed. To collect data for the present study, 2 scales (Beck depression inventory and Alcohol, Smoking, and Substance Involvement screening Test [ASSIST]) was administered to students. Two hundred fifty seven Addis Ababa Institute of Technology final year regular undergraduate students were participated. The results revealed that a high correlation was found between depression and substance abuse. The prevalence of depression is 27.2%. Similarly, the prevalence of alcohol abuse, khat abuse, cigarette abuse and cannabis abuse are 25.5%, 17.7%, 9.5%, and 3.3% respectively. Hence, the overall substance abuse prevalence is 14%. Alcohol is most abused drug followed by khat abuse. Cigarette and cannabis abuse take the 3rd and 4th rank respectively. Multivariate test of significance reveals that gender, religion and the interaction of gender with religion, residence, and ethnicity had an effect on the two combined dependent measures. Females are more depressed than males. In turn, males are more substance abusers than females. The researchers suggested that the university to establish its own substance abuse prevention and treatment working center which is open for psychologists, therapist and other health workers.

## 1. Introduction

Both drug use problems and depression are far more likely to become apparent throughout adolescence. These two conditions are linked to considerable morbidity and death and commonly co-occur in adolescents. Finding the risk factors linked to their co-occurrence is crucial for public health because of the additional financial and psychological strain that the comorbid disease causes.^[1]^ Adolescents or students in higher education have the highest risk of developing severe depressive illness and alcohol and drug dependency compared to the general population.^[1,2]^ Individuals with depression frequently use alcohol and other substances that alter mood as a means of coping with their signs and symptoms. Depression is a common condition among those who are addicted to alcohol or other drugs.^[3]^ This implies people may occasionally turn to drugs as a means of escaping their sadness. In some cases, alcohol or drug use precedes depression, potentially contributing to its development. However, the relationship is complex, and it is not always clear which occurs first.^[4]^

The first focus of this study was depression. One of the most prevalent mental illnesses, depression, has grown to be an increasing worldwide public health concern.^[2]^ Furthermore, depression is acknowledged as a key contributor to the global spread of illness and an essential cause of disabilities.^[5]^ The WHO’s Mental Health Action Plan 2013–2030 additionally highlights the actions required for offering suitable therapy for people with mental diseases, including depression.^[2,5]^ Depression is a condition, a complaint (reported as a symptom), and an experience of emotion (mood state).^[6,7]^ The 3.8% of individuals worldwide were predicted to experience depression in 2023. Depression is one common mental condition that leads to suicide.^[2]^ An estimated 5% of people globally suffer from depression. Depression is quite common in women.^[2,5]^

The prevalence of depression varied in different countries; for example, in Kenya, majority of people reported minimal to no symptoms of depression^[8]^; in Vietnam, 39.6% of people suffer from depression^[9]^; nearly 40% of the students at West Indian University suffer from depression^[2]^; at Brazil Institute medical universities, 12.9%^[10]^; in Karachi, Pakistan, 70%^[11]^ and Australia, 14%^[12]^ of students suffer from depression. According to Sintayehu,^[13]^ 34.5% of people in Adama Town, Ethiopia, were suffered from depression. Research conducted at Ethiopia’s Jimma University also found that 23% of students **were** suffered from depression.^[14]^ The majority of pupils were female.^[11]^ In a similar vein, the prevalence of depressed symptoms is consistent with other research published worldwide.^[15,16]^

The second focus of this study was substance abuse, which is a maladaptive pattern of drug usethat can lead to repeated problems and negative consequences.^[4]^ Almost all nations, both developed and developing, are impacted by the global issue of drug and alcohol abuse. Furthermore, substance abuse affects every aspect of our society; therefore, it must be viewed as a community-wide issue, and everyone has a duty to address it. The most popular drug was alcohol while tobacco was the next most often used drug.^[17]^ Drinking excessively at 1 time or daily are examples of hazardous or problematic behaviors related to alcohol that can be attributed to substance abuse. Substance misuse disrupts your relationships, your family life, and your capacity to work. It might lead to legal problems or risky behaviors like drunk driving.^[18]^ Although statistics indicate that certain people are more prone to use drugs than others, drug abusers come from a variety of backgrounds.^[19]^

There are an estimated 190 million substance addicts worldwide. This number represents 4.3% of the world’s population aged 15 and above, or 3.1% of the total population. This makes it clear that substance misuse, along with associated issues, influences every nation in the world, either directly or indirectly.^[3]^ Substance abuse transcends age, gender, race, and religion.^[20]^ It is common among college students, sex workers, homeless individuals, and youngsters on the streets. According to Fekadu et al,^[21]^ 31%, 26.3%, and 23.3% of students at the College of Medical Sciences in North Western Ethiopia use alcohol, smoke cigarettes, and chew khat, respectively. Furthermore, it is thought that the practice of chewing khat affects a significant portion of Ethiopians, with the productive age group being the most impacted. Chewing khat has detrimental effects on a person’s health as well as social and political difficulties. College and university students consume khat to stay mentally awake and work hard in their academic endeavors but in addition to its negative effects, chewing khat encourages the development of other habits, including cigarette smoking, alcohol consumption, and drug addiction,^[4]^ According to Kebede et al,^[4]^ alcoholism, cigarette smoking, and khat chewing are the most prevalent substance abuse behaviors in Ethiopia and are observed on a daily basis in our communities.

The third focus of the study was the association between substance use and depression. Substance misuse and depression are significantly correlated negatively.^[22]^ Depressed mood was predicted by harmful drinking.^[23,24]^ Depressed students were more likely to have engaged in frequent alcohol use, binge drinking, regular marijuana use and to have used other drugs in the last year.^[25]^ However, there was no significant correlation between drug use and depression.^[8]^

Lastly, the impact of sociodemographic variables on drug addiction and depression was the main emphasis of this study. Depression may affect students of diverse ages, sexes, socioeconomic statuses, religions, and ethnicities since it transcends all social and cultural borders. Significant correlations were found between depression and sociodemographic characteristics, including average income and gender.^[8]^ Major depressive illness is more likely to strike women.^[11,12,26,27]^ However, according to Pesola et al,^[23]^ there were no gender differences in the degree of depression. Students may experience depression due to their academic achievement, relationships with others, and the campus atmosphere.^[28]^ Although statistics indicate that certain people are more prone to use drugs than others, drug abusers come from a variety of backgrounds.^[19]^ For example, men are more likely than women, unmarried than married, live in a city rather than a rural area, and younger than older to consume drugs. Substance abuse is also quite common among prisoners and homeless children.^[19]^

Regarding gaps in previous studies, scholars studied psychoactive drugs in Ethiopia.^[21,29]^ However, the most common drug use and mental health issues in low- and middle-income nations are poorly understood.^[30]^ According to the researcher’s reading and study, Ethiopia has not yet looked at the connection between substance abuse and depression among university students. Adolescents and adults frequently suffer from co-morbid depression and drug misuse.^[31]^ These created financial difficulties for families, individuals, and society at large. Therefore, considering the severe nature of the problem, personal, professional as well and societal interests initiated the current researchers to examine the association of depression and substance abuse in the student population and then recommend to professionals in the area to intervene. Therefore, the research is designed to answer the following questions:

1. What is the prevalence of depression among Addis Ababa Institute of Technology (AAIT) Final year regular undergraduate students?
2. What is the prevalence of substance abuse among AAIT Final year regular undergraduate students?
3. Which substance is most abused by AAIT Final year regular undergraduate students?
4. Is there a [significant association] or co-morbidity between depression and substance abuse among AAIT Final year regular undergraduate students?
5. Is there significant association between socio-demographic characteristics and depression and substance abuse among AAIT Final year regular undergraduate students?

## 2. Theoretical framework

Dual diagnosis and Reward Deficiency Syndrome theories provided support for this investigation. Attempts have been made to elucidate the psychopathological traits of individuals who suffer from dual diagnosis, and the dual diagnosis model summarizes both recent and historical psychopathological issues regarding the comorbidity between mental and substance use disorders, also known as the “dual diagnosis” phenomenon.^[32,33]^ This model places a strong emphasis on social explanations for how mental health and drug use disorders co-occur. Risk factors for substance abuse and depression were similar, including environmental influences and sociodemographic factors that lead to dual diagnoses. Therefore, dual diagnosis theory supports the current study by demonstrating that examining co-occurring disorders simultaneously leads to better outcomes and understanding than examining each condition in isolation.

According to the reward deficiency syndrome idea, some people struggle to feel motivated or pleasure because of a malfunction in their brain’s reward circuits. This is a typical sign of both drug misuse and depression. Depression can exacerbate reward deficiencies, which makes people turn to drugs for fictitious energy increases. Substance misuse can exacerbate depressive symptoms and anhedonia (lack of pleasure) by further harming the brain’s reward system.^[34]^ Because a decrease in the brain’s capacity to naturally experience pleasure leads to a dependence on drugs, which further impairs mood regulation, this study concentrated on the reciprocal effects of depression and substance misuse. Recovery from either condition becomes more difficult as each condition worsens the reward deficiency.

## 3. Methodology

### 3.1. Study design

The study used a cross-sectional survey design, based on a quantitative approach. This was favored as the researchers employed a questionnaire that was given to participants all at once to gather information. The correlational design allows researchers to collect data in one phase.^[35]^ The correlational study design is good for studying organizational contexts when time and resources are restricted and comprehending the connections between the constructs.

### 3.2. Participants

The study was conducted in Addis Ababa University’s Institute of Technology (AAUIT) which is found in Addis Ababa city is the oldest and largest institution of learning. According to AAU registrar statistics report, the total number of regular undergraduate students at AAUIT in 5 batches is 6758. The intention to include only regular undergraduate students was that during the time of data collection, extension, distance and postgraduate students of AAIT were not found at the same place and time.

The study population comprised of 771 final-year undergraduate students from 4 engineering schools. Following the School of Electrical and Computer Engineering (171 total; 37 female, 134 male), the School of Mechanical Engineering (109 total; 20 female, 89 male), and the School of Chemical Engineering (102 total; 17 female, 85 male), the School of Civil Engineering had the most students (389 total; 108 female, 281 male). There were 182 female students (23%) and 589 male students (77%) in the study population. The intention to include only regular undergraduate students was that during the time of data collection, extension, distance and post graduate students of AAIT were not be found at the same place and time.

### 3.3. Sample size and sampling technique

**Figure 1.**
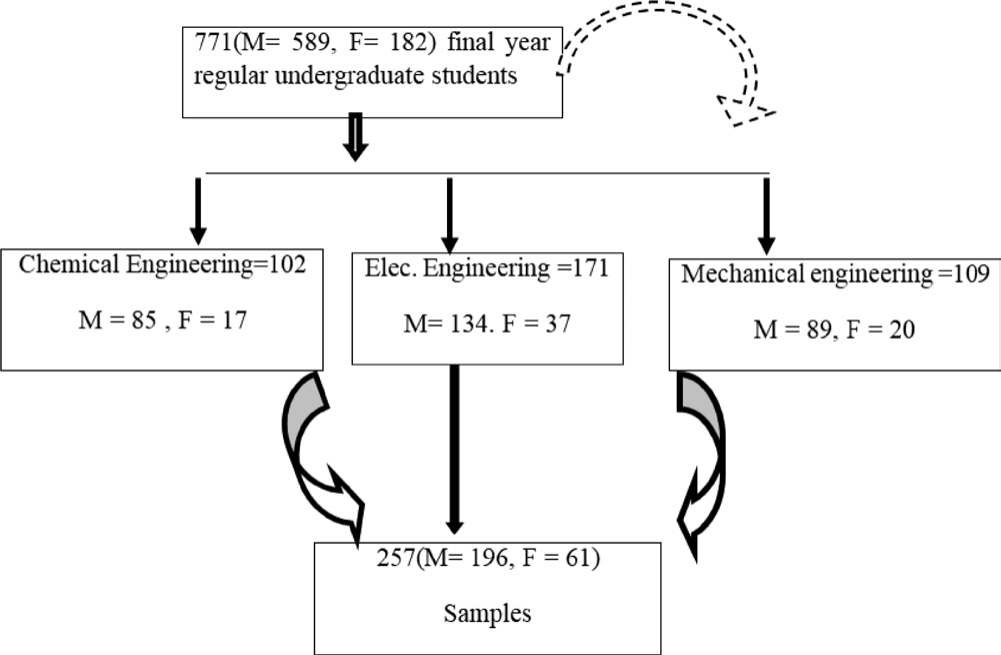
Schematic presentation of sampling procedure. Flow diagram illustrating the selection of final-year undergraduate engineering students for the study. A total of 771 students (M = 589, F = 182) from 3 departments – Chemical Engineering (n = 102; M = 85, F = 17), Electrical Engineering (n = 171; M = 134, F = 37), and Mechanical Engineering (n = 109; M = 89, F = 20) – were initially considered. The final sample comprised 257 students (M = 196, F = 61). Solid arrows indicate the flow of participants from the total pool through each school; the dashed arrow indicates a secondary path considered during recruitment. F = female, M = male, n = total number of participants.

The sample size was determined with the scientific sample size calculator because the technique is very important to get the participants as precisely as needed.^[36]^ Procedurally, the scientific sample size calculator was employed, with a 95% confidence level and a 5% degree of precision, the sample size for 771 tended to be 257 (M = 196, F = 61). A schematic presentation of the sampling procedure is presented below in Figure 1.

The most appropriate sampling method for this study is stratified simple random sampling, as it ensures a proportional representation of gender and school distribution while maintaining randomness. Given that the target population consists of 771 final-year regular undergraduate students across different engineering schools with varying gender compositions, stratification allows for a fair and balanced selection process. The population is first divided into strata based on school and gender, ensuring that each subgroup is adequately represented. Within each stratum, simple random sampling is then applied to select participants, maintaining fairness and eliminating selection bias. This approach results in a final sample of 257 students, consisting of 196 males and 61 females, which accurately reflects the gender ratio of the target population. By using Stratified Simple Random Sampling, the study achieves a more representative and reliable sample, ensuring that findings can be generalized to the broader student population.

### 3.4. Data collection instruments and variables

Predictor variables include: gender, religion, ethnicity, and residence.

Dependent measures contain depression and substance abuse.

Data were collected by structured self-administered questionnaire. It has 3 parts: Demographic information, depression and substance abuse-related items.

### 3.5. Depression

The most popular depression assessment tool, the Beck Depression Inventory (BDI), was created by Beck et al in 1961 and was most recently updated as the BDI-II to conform to the Diagnostic and Statistical Manual of Mental Disorders, Fourth Edition.^[37]^ The psychometric strength of the BDI was evaluated in subsequent research when it was revised, and the BDI-II was introduced in 1996. For a sample of 140 people, the BDI-II’s coefficient alpha was 0.91; for a larger sample of 500 people, it was 0.92.^[37]^ These results imply that the BDI-II exhibits even higher internal consistency than the original version, indicating that it is a very trustworthy instrument for evaluating depression symptoms in both clinical and research contexts. According to reliability coefficients recorded across several versions and research, the BDI and BDI-II are generally recognized as reliable, psychometrically sound tools for assessing depression. The excellent dependability (0.89) found in the current calculation is consistent with earlier results, confirming the BDI-II’s consistency and dependability in psychological assessments. The BDI-II is a well-researched tool with outstanding validity and reliability for assessing depression severity.^[38]^ Therefore, the present study utilized BDI-II.

BDI-II has 21 elements. Each of the 21 elements on the scale included 4 response possibilities that varied in intensity. For each topic, respondents choose one option that best describes their present clinical status “during the last two weeks.” The sum of the scores indicates the degree of depression. Sample items: I feel sad much of the time and I cannot get any pleasure from the thing I used to enjoy. According to this reliability score, the inventory’s components are highly connected and reliably assess the desired construct.

A pilot survey was conducted on 26 students who were not included in the main study but shared comparable backgrounds with the participants in order to assess the reliability, understanding, and total time required for completion. Based on Cronbach’s alpha reliability coefficient, the results most likely offered insightful information about the internal consistency of the survey. If the survey items consistently measured the desired concept, they would have a high- reliability score (usually over 0.7 or 0.8). Furthermore, by identifying any ambiguities or challenges in comprehending the questions, participant input enabled the required changes to be made before the final survey was sent. In the present study, the reliability coefficient for the Beck-II depression inventory, as determined by the computation was 0.89. This indicated that the BDI-II is a very trustworthy instrument for assessing depression symptoms in the present study area.

### 3.6. Substance abuse

To assess substance abuse, Alcohol, Smoking and Substance Involvement Screening Test (ASSIST) was employed. An international team of substance abuse researchers created the ASSIST in 1997 in response to the need for an accurate and trustworthy screening tool for hazardous or problematic substance use that is also culturally sensitive by including 236 participants from 10 locations throughout the globe to participate in a test-retest reliability research.^[39]^ Sample items: in the past 3 months, how often have you used the substances you mentioned (first drug, second drug, etc). The responses were ranged from never once to almost daily. According to the Cronbach coefficient results of the pilot, this coefficient ASSIST scale shows that the test has a high degree of internal consistency. In essence, Cronbach’s alpha assesses whether a scale consistently examines the same underlying concept, in this instance drug use and related behaviors. It does this by measuring how closely related the items. Consequently, the ASSIST may be regarded as a reliable and consistent tool for drug use screening, which makes it appropriate for use in both clinical and research settings.

To sum up, the coefficients of reliability for the 2 scales clearly show that the instruments are highly reliable. However, in the present study, after piloting, some modifications in the instruction were made, some possible choices were added, and some items were rephrased on the socio-demographic data on the way that do not change the meaning which is desired to transfer.

### 3.7. Data analysis and ethical considerations

Following the completion of the questionnaire used to gather the data, its completeness was verified, and any blanks were left unfilled. Version 20 of the Statistical Package for Social Sciences (SPSS) was used for data entry and analysis. Simple descriptive statistics (percentage and frequency) were used to calculate the participants’ demographic information and to determine the prevalence of drug addiction and depression. To examine the association between depression and substance abuse, chi-square test was employed. In addition, by taking gender, religion, ethnicity, and residence as predictor variables and depression and substance abuse as outcome variables, higher order Multiple Analysis of Variance (MANOVA) was performed.

Regarding ethical considerations, first an institutional ethical clearance was obtained from Addis Ababa University’s ethics committee. Then permission was requested from the college dean of AAIT for conducting research and data collection. After weighing the potential benefits, the college dean allowed the researchers to conduct the study. Before data collection, a copy of the approval letter was given to each head of the school to inform them about the study and to ask about their willingness to cooperate in the data collection process. After briefly explaining the purpose of the research and informing the participants that their answers would be kept private, the researchers and 2 assistants administered the pre-made questionnaire to the chosen participants in their classrooms.

## 4. Results

The data which was collected from a total of 243 AAIT final year regular undergraduate students were analyzed to arrive at certain findings. The total number of the distributed questionnaires was 257, of which 243 were filled completely and consistently with a response rate of 94.5%.

### 4.1. Socio-demographic characteristics of respondents

The independent variables of interest in the analysis are gender, religion, ethnicity, and residence. The dependent variables are depression and substance abuse. All the socio- demographic characteristics of respondents are presented in Table 1.

**Table 1:** Socio- demographic characteristics of respondents

Variables		N	%	Variables		N	%
Gender	Male	187	77	Residence friend	Rural	138	56.8
	Female	56	23		Urban	105	43.2
Religion	Orthodox	185	76.1	Ethnic origin	Tigray	47	19.3
	Muslim	32	13.2		Amhara	75	30.9
	Protestant	13	5.3		Oromo	63	25.9
	Catholic	11	4.5		SNNP	35	14.4
	Others*	2	0.80		Others**	23	9.5

* Non-religion.

** Benshangul, Afar and Somalia.

As shown in Table 1, the selected sample consisted of 77% (187 respondents) male and 23% (56 respondents) female, maintaining a proportional gender distribution relative to the target population. The target population comprised 771 final-year regular undergraduate students, of whom 77% (589 students) were male and 23% (182 students) were female. Therefore, the gender proportion in the sample was well-aligned with that of the target population. Regarding residence, 56.8% (138 respondents) live in rural areas, whereas 43.2% (105 respondents) reside in urban areas.

In terms of religion, the majority of respondents, 76.1% (185 individuals), identify as Orthodox Christians. Muslims account for 13.2% (32 respondents), followed by Protestants at 5.3% (13 respondents), Catholics at 4.5% (11 respondents), and others making up 0.8% (2 respondents).

For ethnic origin, the largest group is Amhara, comprising 30.9% (75 respondents), followed by Oromo at 25.9% (63 respondents), Tigray at 19.3% (47 respondents), SNNP at 14.4% (35 respondents), and other ethnic groups accounting for 9.5% (23 respondents). This demographic breakdown indicates a sample that is predominantly male, rural, Orthodox Christian, and Amhara in ethnic composition.

### 4.2. Prevalence of depression and substance abuse

Here below, the prevalence of depression and substance abuse are presented in Table 2.

**Table 2:**
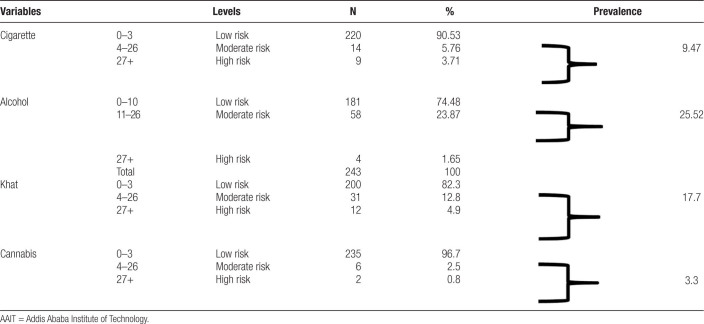
Prevalence of cigarette, alcohol, and khat and cannabis abuse in AAIT final year regular undergraduate students

Those participants who were found at low risk of health due to alcohol and other drugs are considered normal and do not need any intervention, but those who were found in the range of moderate and high risk of health from their pattern of substance use, and may expected experiencing severe problems (such as health, social, financial, legal, relationship) are likely to be taken as abuser. In the above Table 2, it is observed that from current users, 23 (9.47%) fulfilled the criteria of cigarette abuse. Alcohol abusers were 62 (25.52%) followed by khat abuser 43 (17.7%). The least abused drugs in respondents who filled the questionnaire were cannabis 11(4.53%).

**Figure 2.**
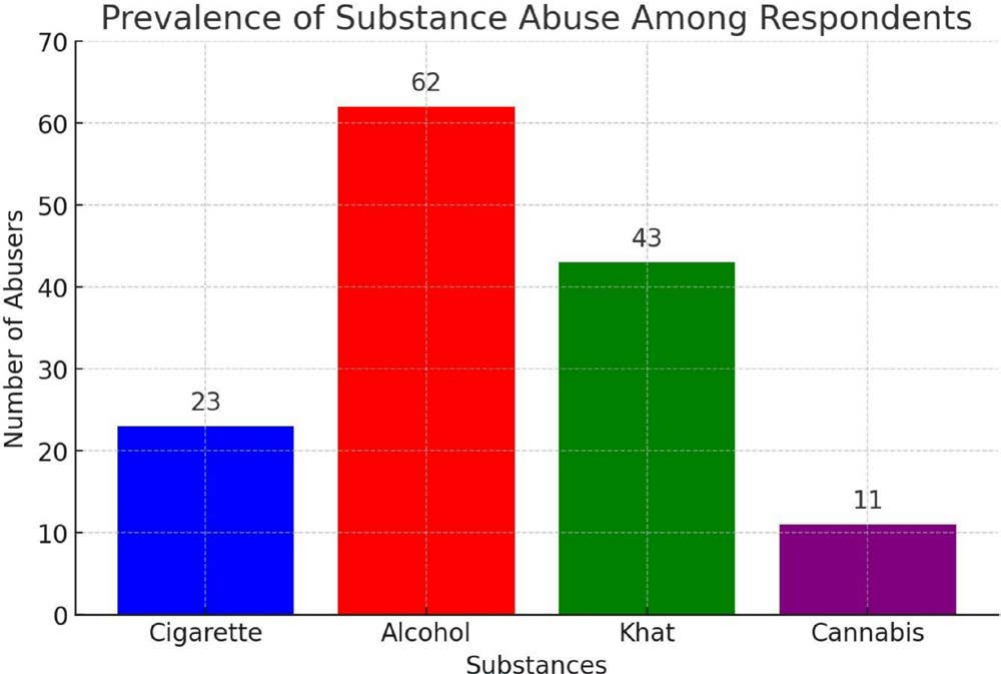
Prevalence of substance abuse among respondents. Bar chart illustrating the prevalence of substance abuse among respondents. The x-axis lists the substances (Cigarette, Alcohol, Khat, and Cannabis), and the y-axis indicates the number of abusers. Alcohol had the highest number of abusers (n = 62), followed by Khat (n = 43), Cigarette (n = 23), and Cannabis (n = 11). n = total number of participants.

As the prevalence rate displayed in Table 2 and Figure 2 above indicate, alcohol is most abused, followed by khat. Cigarette and Cannabis were the least abused drugs which took the third and fourth position respectively.

As shown in Table 3, on 243 students who participated in this study, the overall prevalence of depression was 27.2%. Among those students with depression, a majority (18.5%) had mild depression. According to Beck Depression Inventory (BDI) cut of scores, 72.8% (n = 177) scored as normal (0–13), 18.52% (n = 45) as mild (14–19), 5.8% (n = 14) as moderate (20–28), 2.9% (n = 7) as sever (29–63).

**Table 3:**
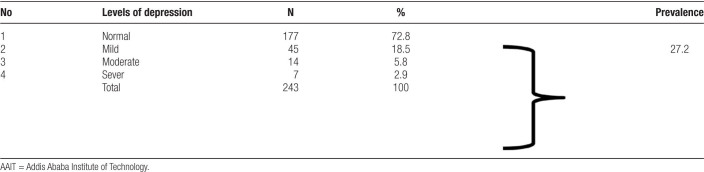
Level of depression and its prevalence in AAIT regular undergraduate students

### 4.3. Co-morbidity of depression and substance abuse

Below the co-morbidity (association) between depression and substance abuse are examined by chi- square test.

As displayed in Table 4 above, 91.1% (112) of respondents who did not abuse any kind of substances were not depressed, and only 8.9% (11) were depressed from non- abused. From participants who were abusing any substances, 54.2% (65) were not depressed, others 45.8 (55%) of participants who were abusing any kind of substances were depressed. Significant differences were observed in depression between substance abused and non-abused (χ^2^(1) = 39.94, *P* = .000).

**Table 4:** The association of depression and substance abuse

Substance	Depression				Total
	Non-depressed		Depressed		
	N	%	N	%	
Non-abused	112	91.1	11	8.9	123
Abused	65	54.2	55	45.8	120
Total	177	72.8	66	27.2	243

### 4.4. Factors associated with depression and substance abuse

The predictor variables undertaken in this study are 4 (4), so to examine the effects of each predictor variables (gender, religion, ethnicity, and residence) on 2 dependent variables (depression and substance abuse). MANOVA were performed and presented as following Table 5.

**Table 5:** MANOVA test between predictor variables (gender, religion, ethnicity, and residence), and dependent measures (depression and substance abuse)

Predictors	Test name		Value	*F*	H. df*	E. df†	Sig.	PES‡
Gender (A)	Depression Substance abuse	Wilks’	0.95	4.70	2.00	192.0	0.010	0.05
Religion (B)	Depression Substance abuse	Wilks’	0.91	2.20	8.00	384.0	0.026	0.04
Ethnicity (C)	Depression Substance abuse	Wilks’	0.93	1.69	8.00	384.0	0.099	0.03
Residence (D)	Depression Substance abuse	Wilks’	0.98	1.56	2.00	192.0	0.212	0.02
A × B	Depression Substance abuse	Wilks’	0.98	.55	6.00	384.0	0.769	0.01
A × C	Depression Substance abuse	Wilks’	0.92	1.98	8.00	384.0	0.047	0.04
A × D	Depression Substance abuse	Wilks’	0.97	3.45	2.00	192.0	0.034	0.04
B × C	Depression Substance abuse	Wilks’	0.91	.74	6.00	384.0	0.825	0.05
B × D	Depression Substance abuse	Wilks’	0.97	1.14	6.00	384.0	0.341	0.02
C × D	Depression Substance abuse	Wilks’	0.93	1.85	8.00	384.0	0.066	0.04
A × B × C	Depression Substance abuse	Wilks’	0.92	4.34	4.00	384.0	0.002	0.04
A × B × D	Depression Substance abuse	Wilks’	0.96	4.33	2.00	192.0	0.014	0.04
A × C × D	Depression Substance abuse	Wilks’	0.95	1.29	8.00	384.0	0.250	0.03
B × C × D	Depression Substance abuse	Wilks’	0.96	1.23	6.00	384.0	0.289	0.02
A × B × C × D	Depression Substance abuse	Wilks’	1.000	–	.000	192.5	–	–

* Hypothesis degree of freedom.

† Error degree of freedom.

‡ Partial eta squared.

As displayed in Table 5 above, the overall MANOVA test show the significant effect of gender on the combined dependent measures, Wilk`s Lambda = .95, *F* (2192) = 4.70, *P < .05, PES* = .05, and religion, Wilk`s Lambda = .91, *F* (8384) = 2.20, *P < .*05, *PES* = .04. Ethnicity and residence were not found statistically significant, *F* (8384) = 1.69, and *F* (2192) = 1.56 respectively as *P > *.05 for both predictor variables.

Further investigations on MANOVA test clearly show that it was not only the main effect gender and religion that had a significant effect on variation of depression and substance abuse. Whereas, the MANOVA table displays a significant interaction effect of gender × ethnicity, Wilk`s Lambda = .92, *F* (8384) = 1.99, *P* < .05, *PES = *.04 on depression and substance abuse. In addition, the interaction effect of gender × residence was significant at Wilk`s Lambda = .97, *F* (2192) = 3.45, *P* < .05, *PES* = .04. Moreover, gender × religion × ethnicity, and gender × religion × residence had significant effect on variation of depression and substance abuse, Wilk`s Lambda = .92, *F* (4, 384) = 4.34, *P* < .05, *PES* = .04, and Wilk`s Lambda = .96, *F* (2, 192) = 4.33, *P* < .05, *PES* = .04 respectively.

The interaction of gender × religion, religion × ethnicity, religion × residence, ethnicity × residence, gender × ethnicity × residence, religion × ethnicity × residence, and gender × religion × ethnicity × residence were found non- significant all at *P* > .05.

In general, multivariate test of significance reveals that gender, religion and the interaction of gender with religion, residence, and ethnicity had an effect on the two combined dependent measures, but this test of significance alone is not enough to explain on which dependent variable, these main effects and interactions had a significant effect. Therefore, mean comparison, univariate ANOVA test performed as follows to see the locus of the statistically significant multivariate test.

From Table 6 above, we can see that gender has significant effect in variation of substance abuse, *F* (1193) = 4.55, *P < *.05, *PES* = .02, and in variation of depressions, *F* (1193) = .05, *P* < .05, *PES* = .00. Religion has also significant effect on depression, *F* (4193) = 2.50, *P* < .05, *PES* = .05, but it was not statistically significant with substance abuse, *F* (4193) = 1.93, *P* > .05.

**Table 6:** Univariate ANOVA test of gender, religion, and interacted predictor variables on each dependent measure

Predictors	DVs	SS	df	MS	*F*	Sig.	PES
Gender (A)	Substance abuse	640.68	1	640.68	4.55	0.034	0.02
	Depression	1.99	1	1.99	0.05	0.05	0.00
Religion (B)	Substance abuse	1084.61	4	271.15	1.927	0.107	0.04
	Depression	438.27	4	109.57	2.501	0.044	0.05
Ethnicity (C)	Substance abuse	1145.76	4	286.44	2.036	0.091	0.04
	Depression	563.67	4	140.92	3.217	0.014	0.06
Residence (D)	Substance abuse	178.83	1	178.83	1.271	0.261	0.01
	Depression	137.46	1	137.46	3.138	0.078	0.02
A × B	Substance abuse	332.58	3	110.86	0.788	0.502	0.01
	Depression	47.35	3	15.78	0.360	0.782	0.01
A × C	Substance abuse	1432.53	4	358.13	2.55	0.041	0.05
	Depression	488.86	4	122.22	2.79	0.028	0.06
A × D	Substance abuse	902.38	1	902.38	6.41	0.012	0.03
	Depression	218.00	1	218.00	4.98	0.027	0.03
B × C	Substance abuse	1211.20	13	93.17	0.66	0.798	0.04
	Depression	707.39	13	54.42	1.24	0.252	0.08
B × D	Substance	470.89	3	156.96	1.12	0.344	0.02
	Depression	289.40	3	96.47	2.20	0.089	0.03
C × D	Substance abuse	614.86	4	153.72	1.09	0.362	0.02
	Depression	131.55	4	32.89	0.751	0.559	0.02
A × B × C	Substance abuse	511.55	2	255.78	1.82	0.165	0.02
	Depression	684.97	2	342.49	7.82	0.001	0.08
A × B × D	Substance abuse	732.04	1	732.04	5.20	0.024	0.03
	Depression	373.14	1	373.14	8.52	0.004	0.04
A × C × D	Substance abuse	402.24	4	100.56	0.715	0.583	0.02
	Depression	57.45	4	14.36	0.328	0.859	0.01
B × C × D	Substance abuse	732.80	3	244.27	1.736	0.161	0.03
	Depression	132.55	3	44.182	1.009	0.390	0.02
A × B × C × D	Substance abuse	0.000	0	–	–	–	0.00
	Depression	0.000	0	–	–	–	0.00

MS = mean squares, PES = partial eta squared, SS = sum of squares.

Gender × Ethnicity had an effect on variation of both substance abusers, *F* (4, 193) = 2.55, *P* < .05, *PES* = .05, and depression, *F* (4, 193) = 2.79, *P* < .05, *PES* = .06. The interaction of gender and religion (A × D) had also significant variation on substance abuse, *F* (1, 193) = 6.41, *P* < .05, *PES = *.03, and on depression, *F* (1193) = 4.98, *P < *.05, *PES* = .03.

Moreover, the interaction of gender, religion, and ethnicity (A × B × C) had an effect on variation of depression only, *F* (2, 193) = 7.82, even at *P* < .001, *PES* = .08, but when gender interact with religion and residence (A × B × D), it affected both substance abuse, *F* (1, 193) = 5.20, *P* < .05, *PES* = .03, and depression, *F* (1, 193) = 8.52, *P* < .05, *PES* = .04.

To identify in which level of the predictor variables were highly depressed and high substance abuser participants were found, group mean comparisons and post- hoc test of comparison were examined as follow:

As observed on Table 7 above, females had statistically significant high depression score (M = 13.32, SE = 1.21) than did males (M = 11.77, SE = .78) with mean difference of 1.55. In addition, statistically significant difference were observed for substance abuse dependent measure, with the average of males (M = 15.39, SE = .1.41) and the average of females (M = 10.56, SE = 2.16) which indicate that males were high substance abuser than females with high mean difference 4.83.

**Table 7:** Group means comparison of gender for the 2 dependent measures

Dependent variables	Gender	Mean	Std. Error
Depression	Male	11.77	0.78
	Female	13.32	1.21
Substance abuse	Male	15.39	1.41
	Female	10.56	2.16

Moreover, to check the contribution of religion to the variation in substance abuse score, the difference of cell mean was tested by using post hoc multiple comparisons (Tukey), and the results of the comparisons of the test suggested that Muslims (M = 15.24, SE = 1.44) were highly depressed than Orthodox (M = 12.45, SE = .73), Pagans (M = 11.00, SE = 4.68), Catholics (M = 10.13, SE = 2.11), and protestants (M = 9.91, SE = 2.04).

The mean comparison for gender and ethnicity interaction revealed that male participants from SNNP had high mean for substance abuse (M = 19.22, SE = 3.22) followed by males from Oromia (M = 17.45, SE = 3.08). Concerning female participants, the mean for abusing substance was higher in female participants from Amhara region (M = 15.69, SE = 4.54) followed by female participants from Oromia (M = 15.60, SE = 4.39) with very little difference. For the second dependent measure that is depression the estimated marginal mean postulated higher mean in female participants of Amhara ethnic origin (M = 17.73, SE = 2.53). The means of females participants of Oromo and SNNP ethnic origin took the second and the third rank (M = 15.35, SE = 2.45) and (M = 14.13, SE = 2.96) respectively. From male sex group, participants of Oromo ethnic origin was highly depressed (M = 13.75, SE = 1.72) than other groups of the same sex.

The gender- residence interaction resulted that both urban males and females participants were high substance abuser (M = 20.31, SE = 2.02), and (M = 11.58, SE = 2.99) respectively than their counter parts rural male participant (M = 11.09, SE = 1.96) rural females (M = 9.54, SE = 2.27). Very high mean difference (MD = 9.22) was observed between urban and rural male participants. The mean of substance abuse dependent measure for rural male was almost equivalent with the mean of urban female participants. The mean for depression in gender and residence interaction was higher in urban females (M = 15.78, SE = 1.67) followed by urban males (M = 14.48, SE = 14.48, SE = 1.13), and rural females (M = 10.87, SE = 1.75). Lower mean (M = 9.40, SE = 1.10) was observed in male participants of rural residence compared to other groups.

The estimated marginal mean of depression in the interaction of 3 predictor variables (gender × religion × ethnicity) was found higher in female’s Muslim religion followers of Amhara ethnic origin (M = 24, SE = 4.68) than male participants from Oromo ethnic origin (M = 21.08, SE = 3.02). Followed these, the mean for depression was higher in female participants of orthodox religion followers among participants of Oromo ethnic origin (M = 16.88, M = 2.14), and male protestant religion follower of Oromo ethnic origin (M = 16, SE = 6.62).

The mean for substance abuse in the interaction of the 3 variables (gender, religion, residence) postulated that abusing substances was higher in male Muslim participants from urban residence (M = 27.46, SE = 4.02) followed by orthodox followers of urban residence (M = 21.18, SE = 1.67). Whereas, in female participants, it was higher in orthodox followers of urban residence (M = 14.57, SE = 2.74) followed by catholic religion followers in urban (M = 12.00, SE = 8.39), and Muslim religion followers in rural residence (M = 11.50, SE = 6.25). For depression as dependent measure, the mean was higher in female participants of catholic religion followers in urban residence (M = 20.41, SE = 2.24).

## 5. Discussion

This study confirmed that numerous mental health conditions, such as a variety of addictions and obsessive and impulsive behaviors, are included in the dual diagnosis and reward deficiency syndrome theories. RDS, also known as an octopus of behavioral dysfunction, is the term used to describe aberrant behavior brought on by a disruption in the neurotransmission cascade of reward due to genetic and epigenetic factors. The major issues in this study were to examine the prevalence of depression and substance abuse. The finding of the study revealed that the prevalence of depression in AAIT final year regular undergraduate students was 27.2%. This is found consistent to be with similar studies in Ethiopia in the previous time. For instance, Yitayal,^[40]^ who conducted a study in Addis Ababa University first year students, reported that the prevalence of depression was 27.7%.The prevalence was greater (23%) than a report from Jimma University,^[14]^ 14% among undergraduate Mekelle University students^[29]^; 19.5% on Baltimore Epidemiologic Catchment Area (ECA),^[41]^ 10.4% in Spanish University students^[11]^ and in Australia, 14%.^[12]^ However, it is remarkably lower than a report from residents of Adama Town, 34.5%,^[13]^ Vietnam 39.6%,^[9]^ 40% of West Indian University students,^[42]^ and 70% of Pakistan students.^[11]^

The study further indicated that alcohol (25.5%) was the most abused drug, followed by khat, Cigarette and Cannabis respectively. In agreement with this, previous researchers found that alcohol was the most abused drugs by students because unlike other drugs, alcohol does not have a severe effect on the health of a person if it is consumed moderately. Concerning the prevalence of substance abuse, the finding of the study is almost consistent with the finding in Mekelle University in 2011, which reported that alcohol was 16.6%, khat was 14.8%, and cigarettez and cannabis were 8.8%.^[29]^ Moreover, in a small survey conducted in Kenya, it was found that khat use was the third most common drug next to alcohol and tobacco.^[43]^ According to Fekadu et al,^[21]^ 31%, 26.3%, and 23.3% of students at the College of Medical Sciences in North Western Ethiopia use alcohol, smoke cigarettes and chew khat, respectively. In similar vein, according to Kebede et al^[4]^ (2005), alcoholism, cigarette smoking, and khat chewing are the most prevalent substance abuse behaviors in Ethiopia and are observed on a daily basis in our communities. A study conducted on Jimma University showed that 24.8% students were regular chewers.^[14]^ In Uganda, the use of khat among students, transporters, and law enforcement officials were 9.2%, 68.8%, and 97.1% respectively.^[44]^

The present study also revealed that substance abuse and depression were correlated. In line with the present study, prior scholars found that substance abuse and depression are significantly correlated.^[22–24,45–48]^ Depressed students were more likely to have engaged in frequent alcohol use, binge drinking, regular marijuana use, and to have used other drugs in the last year.^[25]^ Contrary to the present study, Shah et al^[8]^ found that there was no significant correlation between drug use and depression.

Lastly, the multivariate test of significance reveals that gender, religion and the interaction of gender with religion, residence, and ethnicity affected the two combined dependent measures. Females are more depressed than males while males are more substance abusers than females. In congruent with the present study, significant correlations were found between depression and sociodemographic characteristics, including gender.^[8,19]^ Major depressive illness is more likely to strike women.^[11,12,27]^ Contrary to the present study, according to Pesola et al,^[23]^ there were no gender differences in the degree of depression.

## 6. Conclusion, implications, limitations, and suggestions for future scholars

The prevalence of depression was 27.2% whereas, 14% for substance abuse. Alcohol was most abused in AAIT final year regular undergraduate students. There were high association between depression and substance abuse. Depression was higher in those participants who were abusing substances. Compared with other drugs, depression was higher in participants who abused tobacco. The most often misused substance is alcohol, which is followed by khat. Abuse of cigarettes and cannabis comes in third and fourth, respectively. The two combined dependent variables were impacted by gender, religion, and the interaction of gender with religion, residency, and ethnicity, according to the results of the multivariate test of significance. Compared to men, women are more depressed. In turn, men misuse substances at a higher rate than women.

This finding has theoretical and practical implications. First, theoretically, this research adds theoretical evidence to the existing literature on the depression and substance abuse by revealing the empirical relationship between students’ substance usage and depression. Second, the results have important managerial implications for comprehending and supervising variables that might result in higher students’ depression levels. This suggests that: For better effect, the university should work on empowering professionals who work on students’ health at university such as therapists, and psychologists assign them by establishing it its own working center. As a result, these professional in the area will work on regular counseling and facilitate groups’ therapy especially for individuals who are identified at risk, so through experience sharing, they can learn each other. By giving great attention for the degree of substance abuse and depression problems among university students, health workers should take the initiative to work on improving the prevention of students` substance abuse and depression by focusing on giving information about orientation for life.

When evaluating these findings, it is important to take into account a number of research limitations. First off, our results might not be generalizable to other groups because the sample consisted mostly of Addis Ababa University students. Second, we used self-report as our measure of substance use, which may have limited the accuracy of the information gathered about substance use behaviors. However, other studies have indicated that rather than gathering information directly from friends, adolescents’ opinions of their friends’ antisocial conduct are a better indicator of their involvement in risky behavior.^[49]^ To find out if these results change depending on how big an adolescent’s buddy network is, future research might look at larger friend networks. Third, extrapolations regarding causal linkages are limited by the cross-sectional methodology used in this investigation. Future interested researchers are recommended to conduct a longitudinal study in assessing the relationship between depression and substance abuse in university students’ population starting from student’s entry of the university and their experimentation of drugs to their habitual use in accordance with depression variation.

## Acknowledgments

We thank the participants for participating in this research.

## Author contributions

**Conceptualization:** Kelemu Zelalem Berhanu.

**Data curation:** Kefie Manaye Mengistie.

**Formal analysis:** Kefie Manaye Mengistie.

**Supervision:** Kefie Manaye Mengistie, Kelemu Zelalem Berhanu.

**Validation:** Kefie Manaye Mengistie.

**Writing – original draft:** Kefie Manaye Mengistie, Kelemu Zelalem Berhanu.

**Writing – review & editing:** Kefie Manaye Mengistie, Kelemu Zelalem Berhanu.
